# A quantitative study on factors influencing enrolment of dairy farmers in a community health insurance scheme

**DOI:** 10.1186/s12913-016-1925-1

**Published:** 2016-12-09

**Authors:** Tineke de Groot-de Greef, Lydia V. Monareng, Janetta H Roos

**Affiliations:** 1 Department of Health Studies, University of South Africa, P.O. Box 392, Unisa 0003, Pretoria, South Africa; 2 Christian University of Applied Sciences, Ede, Netherlands

**Keywords:** Access to community health insurance, Dairy farmers, Enrolment status, Information provision

## Abstract

**Background:**

Access to affordable and effective health care is a challenge in low- and middle- income countries. Out-of-pocket expenditure for health care is a major cause of impoverishment. One way to facilitate access and overcome catastrophic expenditure is through a health insurance mechanism, whereby risks are shared and financial inputs pooled by way of contributions. This study examined factors that influenced the enrolment status of dairy farmers in Western Kenya to a community health insurance (CHI) scheme.

**Methods:**

Quantitative, cross-sectional research was used to describe factors influencing the enrolment in the CHI scheme. Quota and convenience sampling was used, recruiting a sample of 135 farmers who supply milk to a dairy cooperation. Data were collected using a structured interview schedule and analysed using Stata SE, Data Analysis and Statistical Software, Version 12.

**Results:**

Factors influencing non-enrolment were identified as affordability (40%; *n* = 47), unfamiliarity with the management of the scheme (37%; *n* = 44) and a lack of understanding about the scheme (41%; *n* = 48). An exploratory factor analysis was used to reduce the variables to two factors: information provision and understanding community health insurance (CHI). Logistic regression identified factors associated with enrolment in the Tanykina Community Healthcare Plan (TCHP). Supplies of less than six litres of milk per day (OR: 0.22; 95% CI: 0.06–0.84) and information provision (OR: 8.77; 95% CI: 2.25–34.16) were significantly associated with enrolment in the TCHP.

Nearly 30% (29.6%; *n* = 40) of the respondents remarked that TCHP is expensive and 17% (*n* = 23) asked for more education on CHI and TCHP in an open-ended question.

**Conclusion:**

Recommendations related to marketing strategies, financial approach, information provision and further research were outlined to be made to the management of the TCHP as well as to those involved in public health.

**Electronic supplementary material:**

The online version of this article (doi:10.1186/s12913-016-1925-1) contains supplementary material, which is available to authorized users.

## Background

In 2005 the World Health Assembly of the World Health Organization (WHO) adopted a resolution on sustainable health financing, universal coverage and social health insurance, indicating that health-financing systems in many countries need to be further developed in order to guarantee access to health services. Moreover, prepayment and pooling of resources and risks are basic principles in financial risk protection; and the choice of a health financing system should be made within the particular context of each country [1]. This resolution addressed a major problem in Africa, namely out-of-pocket payments for health care services. Almost 60% of health care in Africa is paid by patients out of their pockets, causing many to fall into a poverty trap [1, 2]. Therefore, it is a challenge to move away from out-of-pocket payments towards some form of prepayment and ultimately universal coverage. It is in the transition process towards universal coverage, where a community health insurance (CHI) scheme plays a vital role in protecting as many people as possible [3].

CHI refers to voluntary health insurance schemes organised at community level. CHI is not a new concept, having emerged in developing countries in the second half of the 1980s as an attractive and powerful method to secure better access to health services and greater financial protection for poor populations [3]. Despite the evidence on the potential of CHI to improve access to quality health care, enrolment in sub-Saharan Africa remains low [4].

There is strong evidence that CHI improves the mobilisation of resources for health and the utilisation of health services [5]. It also provides financial protection for members in terms of reducing their out-of-pocket expenditure [5].

The last two decades have seen an apparent boom in CHI in sub-Saharan Africa. In 2004 estimates already indicated the existence of approximately 900 schemes in sub-Saharan Africa [4]. However currently, across sub-Saharan Africa, CHI is beleaguered by low enrolment. Apart from a few isolated successes such as in Ghana and Rwanda, reviews consistently report enrolment rates of between 1% and 10%. This low enrolment rate has become a source of interest for researchers [4]. The main focus is to investigate the impact that CHI has on access to care, financial protection against the cost of illness, quality of care and its contribution to the performance of health financing systems. However, there is little evidence of the impact of CHI on community empowerment, meaning the impact on social inclusion and utilisation patterns among vulnerable groups [5]. An analytical review of the existing literature on the major operational difficulties hampering CHI development in sub-Saharan Africa was conducted. One of the five categories that emerged in research was low enrolment rates. Recommendation was further made that an empirical investigation about those issues that seem to discourage people from joining CHI be conducted [5].

Middle- to low income-countries, including Kenya, are faced with the challenges as described to reform their health systems in such a way that it promotes equity and efficiency [6]. It is estimated that in 2012/2013 the contributions of the three main role players to the total health expenditure (THE) in Kenya was as follows: government (34%), private households (40%) and donors (26%) [7].

The formal dairy industry is regarded as the fastest-growing agricultural sub-sector in Kenya. Unfortunately smallholder farmers do not see dairy as a business, leading to a lack of investment in good feeds and animal health and low production. These farmers sell an estimated 3–5 l milk per day, while it is estimated that a production of 15 l per day is required to bring the family over the poverty line [8]. The farmers fall within the lower socio-economic status (SES) groups of Kenya’s population with an average monthly income of around Kenyan Shilling (KSh) 10 000 per household [9] .

In Kenya, 36% of healthcare is paid by patients out of their pockets, causing many to fall into a poverty trap [7]. The challenge therefore is to move away from out-of-pocket payments towards some form of prepayment. The estimated health coverage in Kenya, comprising both voluntary and mandatory insurance schemes is very low (10%). It seems to be skewed to the urban population (19.7%) compared to the rural population (7.4%), and the richer part of the population (26.4%) compared to the poorest part (1.9%) [6].

TCHP is a joint venture between TDPL (owner), AAR ([African Air Rescue] [medical insurance provider]) and PharmAccess Foundation (implementer) [10]. They did their best to involve the community and develop an attractive benefit package, to improve healthcare access and financially protect as many people as possible towards healthcare provision [3]. The TCHP deducts the cost from members’ milk account at the end of the month to pay for coverage. The healthcare plan has different options, starting with KSh375 for one person to a maximum coverage for a family of seven and more for KSh1, 499 [11]. The mean price of milk in 2008 received by a farmer is KSh29.30 [12]. This means that farmers should deliver at least 1.7 l milk daily, just to cover their monthly premium for maximum coverage.

TCHP members have access to quality health care, provided by qualified medical staff at upgraded clinics and hospitals, with available medication. Other benefits include referral to two quality referral facilities and covers transport in emergency cases [11]. On first inspection, TCHP seemed to be a very well-constructed CHI, as it was evidence-based, as well as tailored to the needs of the community. One and a half years after the official launch of the programme in 2011, 477 members out of a monthly average of 3 366 active Tanykina Dairy Plant Ltd(TDPL) members along with their families, were enrolled in the TCHP [10]. The low enrolment figures triggered an interest to empirically investigate the situation. What factors influence enrolment of dairy farmers in a CHI for better access to health care?

### Objective

The objective of this research was to describe factors influencing enrolment by dairy farmers in TCHP.

## Methods

A quantitative cross-sectional research design was used to describe factors influencing the enrolment of dairy farmers into the health insurance scheme of the TCHP. The study population comprised all dairy farmers who supplied milk to four main collection centres of TDPL in August 2012 (Table 1). Although there were four locations of milk collection, for convenience purpose the sample for this study was drawn from dairy farmers who supplied milk to Lemook collection centre. The sample size was determined by considering the monthly average of 3 366 milk suppliers of whom 14% was enrolled as TCHP members. The sample size formula used to estimate the sample size for this study is as follows: ${n}^0=p\left(1-p\right){\left(\frac{z}{E}\right)}^2$.

**Table 1:** Population per location

	Enrolment status					
Location	Non-enrolled	Enrolled	Active	Suspended^a^	Terminated^a^	Total
Salien	846	156	137	12	7	1002
Lemook	215	66	47	11	8	281
Sangalo	430	166	19	12	7	596
Surungai	466	89	35	5	3	555
Total	1957	477	238	40	25	2434

^a^These respondents (suspended and terminated) were included in the status of enrolled as they had experience of being enrolled to TCHP

The 477 enrolled members to the TCHP represent 14% of the average active TDPL members (*n* = 3 366). The researchers therefore decided that from the selected sample, a quota of 14% should be enrolled to the TCHP scheme. Quota sampling was used to select 135 respondents to ensure that none of the enrolled or non-enrolled groups to the TCHP is overrepresented or underrepresented. The quota was calculated by taking the average number of milk suppliers over a 12-month period (September 2011–August 2012). There was much fluctuation in the number of milk suppliers due to seasonal changes (Additional file 1: Table S1).

The proportion of respondents enrolled to the CHI scheme versus respondents who were not enrolled were determined through quota sampling, while convenience sampling was used to select the respondents.

Only active TDPL members who supplied milk in August 2012 to Lemook milk collection centre were included. These members were 18 years and older, including both males and females who could speak sufficient Kiswahili or English. The study included both enrolled and non-enrolled members of the CHI.

### Structured interview guide

A structured interview schedule was developed by the researchers and was translated from English into Kiswahili (the national language of Kenya) and was used together with the English interview schedule to collect data

The structured interview guide comprises socio-demographic questions about gender, age, marital status, number of children, education and income as independent variables (Table 2) and enrolment status to TCHP as the dependent variable. The questions were mainly closed-ended. Sixteen questions relating to TCHP were asked in Likert format (using a 5-point scale running from “strongly agree” through to “strongly disagree”). These include knowledge and understanding of the TCHP, the benefit package, affordability and payment modalities, management of the TCHP, accessibility of health services and attitude towards CHI, understanding of risk pooling, socio-cultural practices and quality of health services (see Table 3). Both the “average milk supply per day” and the variable: “another income source in addition to milk” was used in this research as socio-economic status (SES) indicators.

**Table 2:** Background characteristics of the respondents

Variable	Number	%
Gender (*n* = 135)		
Male	73	54.0
Female	62	46.0
Age (*n* = 135)		
18–24 year	3	2.2
25–34 year	28	20.7
35–44 year	44	32.6
45–54 year	24	17.8
55–64 year	20	14.8
65 year and older	16	11.9
Marital status (*n* = 135)		
Married	118	87.4
Never been married	9	6.7
Divorced/Separated/Widowed	8	5.9
Number of children (*n* = 134)		
0–2	24	17.9
3–4	47	35.1
5–6	34	25.4
7–8	16	11.9
9 and more	13	9.7
Education level (*n* = 135)		
None	38	28.1
Primary	44	32.6
Secondary	39	28.9
Post- secondary	14	10.4

**Table 3:** Respondents' level of agreement on statements about TCHP and CHI (*N* = 135)

Statement	Enrolled in n (%) *n* = 17			Non-enrolled in n (%) *n* = 118		
	Disagree	Undecided	Agree	Disagree	Undecided	Agree
1. I know what TCHP is.	0	0	17 (100)	27 (23)	7 (6)	84 (71)
2. I understand how TCHP works.	0	3 (18)	14 (82)	48 (41)	7 (6)	63 (53)
3. I know what is included in the benefit package of TCHP.	1 (6)	1 (6)	15 (88)	39 (33)	21 (18)	57 (48)
4. The benefit package of TCHP is satisfactory.	0	2 (12)	15 (88)	29 (24)	32 (27)	57 (49)
5. TCHP is affordable.	3 (18)	0	14 (82)	47 (40)	28 (24)	43 (36)
6. Paying premium via milk is an attractive payment method.	2 (12)	1 (6)	14 (82)	13 (11)	15 (13)	90 (76)
7. TCHP is well promoted in the community.	2 (12)	1 (6)	14 (82)	18 (16)	21 (18)	99 (66)
8. People talk positively about TCHP.	1 (6)	6 (35)	10 (59)	8 (7)	26 (22)	84 (71)
9. I know how TCHP is set up and managed.	3 (18)	1 (6)	13 (76)	44 (37)	33 (28)	41 (35)
10. I trust the TCHP management.	1 (6)	3 (18)	13 (76)	20 (17)	41 (35)	57 (48)
11. Health insurance helps people to prevent financial disaster.	1 (6)	1 (6)	15 (88)	2 (2)	4 (3)	112 (95)
12. Health insurance is useful for my family.	0	1 (6)	16 (94)	1 (1)	7 (6)	109 (93)
13. I don’t mind contributing money to a healthcare plan and not benefit from it while others do.	2 (12)	0	15 (88)	15 (13)	14 (12)	88 (75)
14. In the last 12 months my household had to pay a lot of money for healthcare and medication.	13 (76)	0	4 (24)	59 (50)	2 (2)	57 (48)
15. I would rather wait and see whether TCHP is a good plan before I enrol.	13 (82)	2 (12)	1 (6)	30 (26)	24 (21)	63 (53)
16. If I become really sick, the community will do a “harambee”, so I don’t need a health insurance.	13 (76)	2 (12)	2 (12)	96 (83)	8 (7)	12 (10)

### Data collection

The first author was employed at Moi University in Eldoret from 2007 till 2012 during the time of the research at TDPL), where all the members of TDPL have been offered a CHI called the Tanykina Community Healthcare Plan (TCHP).

The first author and three trained research assistants collected data in September 2012 over a period of three days. The first day of data collection was at the cattle market of Lemook, where 61 interviews took place. The other 74 interviews took place at the homesteads of the respondents, who were selected by means of convenience sampling.

### Data analysis

The analysis strategy designed for the study was aimed at attending to the research objectives.

It also included using descriptive and inferential statistics by means of the Stata SE and Statistical Software, Version 12 for Windows. Descriptive statistics were used to describe and summarise data and the results were presented in the form of percentages, frequencies, tables, bar graphs and pie charts. Inferential statistics were used to identify relationships between and among variables. Exploratory factor analysis was used to reduce the multidimensionality of the data as findings on factors related to enrolment were of paramount significance to issues of health-care access for the dairy farmers. In this study, factor analysis was conducted on all 16 variables contained in the Likert scale to examine interrelationships among large numbers of variables and disentangle those relationships to identify clusters of variables that are most closely linked together. These clusters of variables were called factors [13]. The first author together with a statistician examined the eigenvalues to decide how many factors would be included in the factor analysis.

Analysis was done at a significance level of 5%, at a *p* value of 0.05 (at a 95% confidence level) and one degree of freedom. Communality (h^2^) is the squared multiple regression coefficient for each variable. Thus, the communality coefficient described the amount of variance in a single variable explained across all the factors in the analysis [13]. The communality for a variable was obtained by summing the squared factor loadings on the variable for each factor.

Logistic regression was conducted to predict the potential of enrolment in the TCHP.

The Cronbach’s co-efficient reliability of the instrument was done to ensure reliability [14]. Cronbach’s co-efficient reliability results were 0.85 and therefore greater than 0.7 which indicated internal consistency reliability [15].

## Results

The respondents’ socio-demographic data included gender, age, marital status, number of children, education level, milk supply and distance to nearest TCHP Health Centre. The results are displayed in Table 2.

The results indicate that the average age of those enrolled tended to be higher than that of those who were not enrolled. Out of the enrolled respondents 38% (*n* = 6) had seven or more children, compared to the non-enrolled 19% (*n* = 33).

Of those enrolled in TCHP, 47% (*n* = 8) indicated not having completed primary education, 23% (*n* = 4) indicated having completed primary education, 18% (*n* = 3) indicated having completed secondary education and 12% (*n* = 2) indicated having completed tertiary education.

The Chi-square analysis was done to establish if there was a significant difference between enrolled and non-enrolled respondents and their educational status, gender, marital status, number of children, age and distance to the nearest healthcare facility. The results of all these variables respectively are not significant at *p* < 0.05.

**Fig. 1:**
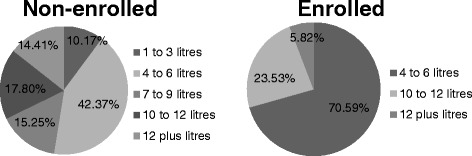
Milk supply in litres per day by enrolment status (*N* = 135)

The average milk supply per day differed per enrolment status of the respondents, as depicted in Fig. 1.

Of the enrolled respondents, 71% (*n* = 12) supplied an average of 4–6 l, 24% (*n* = 4) supplied 10–12 l, and 6% (*n* = 1) supplied more than 12 l per day. Of the non-enrolled respondents, 42% (*n* = 50) supplied an average of 4–6 l, 18% (*n* = 21) supplied 10–12 l, and 15% (*n* = 18) supplied 7–9 l, with 15% (*n* = 17) supplying more than 12 l and 10% (*n* = 12) supplying only 1–3 l per day.

Out of the enrolled respondents, 35% (*n* = 6) supplied milk for 10–12 months during the past year, while for the non-enrolled, 27% (*n* = 32) supplied milk for 10–12 months.

Of the 135 respondents, 89% (*n* = 120) indicated that they have an alternative source of income to that of the milk supply, enabling the majority to afford the TCHP premium.

Out of the 135 respondents, 24% (*n* = 33) indicated having other health insurance than TCHP. Of the respondents, 29% (*n* =5) of the TCHP enrolled indicated that they also belong to another health insurance scheme to complement TCHP.

Out of 131 respondents, 34% (*n* = 45) indicated that they travelled at most 30 min to the nearest TCHP Health Centre, while 45% (*n* = 59) of these respondents indicated travelling between 30 and 60 min and 21% (*n* = 27) travelled for more than one hour. Data from four respondents was missing. It is possible to speculate that they did not know of a health centre nearby and therefore could not indicate the distance. Enrolled respondents tended to live closer to the TCHP facilities, since 41% (*n* = 7) travelled less than 30 min to these facilities, as compared to the non-enrolled, where only 33% (*n* = 38) travelled less than 30 min.

Respondents were asked about their agreement or not on statements relating to enrolment with TCHP and CHI on a five-point Likert scale. The response alternatives of “strongly agree” and “agree" were grouped together as “agree”, while the response alternatives of “strongly disagree” and “disagree” were grouped together as “disagree”, while the undecided responses remain unchanged. The results are displayed in Table 3.

The following was concluded based on Table 3 regarding the level of agreement on the statements:

Of the 17 enrolled respondents, 82% (*n* = 14) agreed that the TCHP was affordable for them. This indicates a positive experience of the price/quality ratio for those enrolled in the TCHP. However, among the non-enrolled, 36% (*n* = 43) agreed and 40% (*n* = 47) disagreed that TCHP is affordable. This means that affordability may indeed constitute a major threshold for enrolment. Of those enrolled, 82% (*n* = 14) and 76% (*n* = 90) of the non-enrolled agreed that paying by means of milk supplied was an attractive method of meeting their premiums. This means that paying premiums via the milk account is, overall, considered an attractive method of payment for the members of the TDPL.

Of the enrolled respondents, 76% (*n* = 13) agreed that they know how the TCHP is set up and managed and that they trust the management. This indicates that for the TCHP members, it is clear how the TCHP is managed, indicating a high level of trust. For the non-enrolled, the management structure is not clear as 37% (*n* = 44) of the respondents disagreed and 28% (*n* = 33) were undecided. However, 48% (*n* = 57) agreed that they trust the TCHP management, despite the high percentage of respondents who attest to not being familiar with the management.

A high percentage of the enrolled (88%; *n* = 15) and the non-enrolled (95%; *n* = 112) agreed that health insurance helps people to prevent financial disaster. A high percentage also agreed that health insurance is useful for their family (enrolled 94%; *n* = 16, and non-enrolled 93%; *n* = 109). This indicated that there is a positive attitude towards CHI among the TDPL farmers and that they tend to understand the basic principle of health insurance.

However, the majority of the respondents (83%; *n* = 96 of non-enrolled; 76%; *n* = 13 of enrolled) did not agree that they would hold a “harambee” in the case of serious illness. A “harambee” refers to communal fundraising in case of high health expenditures on the part of a member of the community [16].

Factor analysis was an appropriate method for reducing the multidimensionality of variables measured on a Likert scale [17]. A correlation matrix of the scores (Table 4) was developed. Table 4 shows the nine remaining variables’ correlation of more than 0.3 in absolute value, where the other seven variables with a correlation of less than 0.3 were omitted.

**Table 4:** Correlation matrix of the remaining variables

Variable	V1	V2	V3	V4	V6	V9	V10	V11	V12
V1	1								
V2	-0.3225	1							
V3	-0.1369	-0.6136	1						
V4	-0.2832	0.0111	-0.2546	1					
V6	0.2027	-0.0425	-0.0373	-0.3832	1				
V9	0.0878	-0.1005	-0.1273	-0.1118	-0.0388	1			
V10	0.0387	-0.1329	0.039	-0.1964	-0.1432	-0.3705	1		
V11	-0.0592	0.0515	-0.0738	0.1628	-0.1033	0.0094	-0.1482	1	
V12	0.0832	-0.0575	0.0622	-0.0553	0.0886	-0.1185	0.0547	-0.6241	1

The overall Keiser-Meyer-Olkin (KMO) measure of sampling adequacy was 0.813. This confirmed that factor analysis was appropriate to reduce the multidimensionality of the data set.

By following the steps of exploratory factor analysis namely the scree test and factor rotation, two factors were isolated as demonstrated in Table 5. A factor loading is actually the regression coefficient of the variable on the factor. The factor loading indicates the extent to which the single variable is related to the cluster of variables [13]. In variable 1, the factor loading is 0.75 for factor I and -0.04 for factor II. Squaring the factor loadings ([0.75]^2^ = 0.5625, and [-0.04]^2^ = 0.0016 gave the amount of variance in variable 1, which explained factors I and II.

**Table 5:** Factor loadings for two factors

Variable	Statement	Factor I	Factor II	Uniqueness= 1-(h)^2^= 1-communality
V1	I know what TCHP is	0.75	-0.04	0.43
V2	I understand how TCHP works	0.91	0.04	0.16
V3	I know what is included in the benefit package of TCHP	0.90	0.03	0.19
V11	I know what is included in the benefit package of TCHP	0.05	0.72	0.48
V12	Health insurance is useful for my family	-0.00	0.72	0.48

In Table 5, the communality coefficient for variable 1 was (0.75)^2^ + (-0.04)^2^ = 0.56.

Uniqueness is the variance that is unique for each variable and not shared with other variables. It is equal to 1 minus communality (variance that is shared with other variables). For example, 48% of the variance in “Health insurance helps people prevent financial disaster” is not shared with other variables in the overall factor model. On the contrary, “I understand how TCHP works” has a low variance not shared with other variables (16%). The greater the “uniqueness” (uniqueness > 50%), the lower the relevance of the variable in the factor model. Table 5 does not have a variable with uniqueness greater than 50%, showing the high relevance of the variables in the factor model [14].

Table 5 showed that the first three measured correlated variables explain over 50% of the variability in factor I, while the last two explain over 50% of the variability in factor II. Thus, there were two uncorrelated latent variables explaining two different characteristics of the data.

The next step was to name these factors in order to identify the broad construct of meaning that has caused these particular variables to be so strongly inter-correlated [13]. Due to the meaning captured by the variables, the researchers decided to term factor I: “information provision” and factor II: “understanding CHI” – see Table 6.

**Table 6:** Factor variance

Factor	Variance	Difference	Proportion	Cumulative
Information provision	2.21	1.18	0.77	0.77
Understanding CHI	1.03	-	0.36	1.13

The two factors now explained the 16 variables and were manageable in the subsequent analysis. The predicted scores “information provision” and “understanding CHI” were uncorrelated, and each had a mean of 0.

A multivariable regression analysis was conducted in order to predict the chance of enrolment in the TCHP into its odds. In Table 7, the two factors “information provision” and “understanding CHI”, the quantity of supplied milk, and visits by sales executives were included as covariates in the logistic regression model, where OR stands for odds ratio and CI for confidence interval.

**Table 7:** Logistic regression model

	Adjusted	Unadjusted
Enrolled	OR (95% CI)	OR (95% CI)
Milk (>6 vs. <=6)	0.22 (0.06–0.84)	0.46 (0.15–1.39)
Information provision	8.77 (2.25–34.16)	5.05 (1.92–13.30)
Understanding CHI	0.45 (0.19–1.09)	0.87 (0.46–1.65)
Sales executive visit (yes vs. no)	4.9 (0.84–28.82)	0.14 (0.03–0.64)

The adjusted effect of the quantity of daily milk supply as seen in Table 7 indicates a strong association with enrolment (OR: 0.22; 95% CI: 0.06–0.84). Farmers supplying more than six litres milk per day were 78% less likely to be enrolled compared to those supplying less than six litres per day. Information provision was significantly associated with enrolment in TCHP (OR; 8.77; 95% CI: 2.25–34.16) where an increase in information provision also predict an increase in the odds of enrolment.

An open question, “Do you have any comments you would like to make about TCHP?” was asked. Of the 134 responses received, 29.6% (*n* = 40) remarked that TCHP was expensive while 3% (*n* = 4) responded that TCHP is expensive and far. Seventeen per cent (*n* = 23) suggested that more education is needed and 8% (*n* =11) indicated that the community members need to be sensitised. Other responses made were that the TCHP should continue with their good work (4.5%; *n* = 6), it is far (3.8%; *n* = 5), it is good (5.2%; *n* = 7), it is convenient (2.2%; *n* = 3) and need promotion (2.2%; *n* = 3).

## Discussion

The objective of this research was to describe factors influencing enrolment by dairy farmers in TCHP which could assist TCHP to improve the implementation of the CHI.

Overall there were slightly more male (54%) than female respondents, but of the enrolled respondents 71% were male. The male respondents were mainly interviewed at the cattle market, and the females at their homesteads. Findings in a previous qualitative study [18] conducted among TDPL members found that both men and women take decisions on health care in the target population. “Although the man takes the final decision, the woman of the house has an influential role. She is the one who is aware of the health issues of the entire family and takes care of the children when they are ill. She will come with advice to the husband” [19].

The average age of the respondents enrolled to the TCHP was higher than those who were not enrolled, although this difference was not significant. The enrolment of older people to a CHI is described as adverse selection, whereby those older or in a more fragile state of health are more likely to subscribe to health insurance than younger and healthier people [20] . Adverse selection is an important concern, especially for subsidised CHIs [21].

Married respondents made out 82% (*n* =14) of the TCHP enrolled members. This was supported by a study in Nigeria which found that married couples have a higher tendency to subscribe to the National Health Insurance (NHI) than persons who were not married [22]. Formerly married members of the community as well as those who have never been married were seen as less likely to participate in a public health insurance programme [23].

Enrolment in a CHI was associated with a higher proportion of children in the household [24].

The findings of this study however indicated that there is no significant difference between enrolment status and the number of children in a household.

Previous studies indicated that a higher level of education had been associated with enrolment in a CHI [24]. In one study it was noted that education “above that of primary education” is a significant determinant for enrolment in a CHI [25]. This appears logical, since highly educated people tend to better understand the underlying principles of health insurance and to better budget their income. This was confirmed by findings from a study conducted in 2010 concerning the degree of literacy, whereby a low degree of literacy was associated with poor households and low coverage of a CHI. Results of this study on the contrary did not find a significance difference between enrolment and educational level.

Other studies confirm that poor people are less likely to enrol in a CHI, coining the term “exclusion effect” to describe the phenomenon [26–28]. Since the majority of the TCHP members pay their premium via their milk account, it was interesting to know if the respondents were able to supply their milk continuously throughout the year. During the time of the research the mean milk price received by farmers was KSh29.30 per litre. A farmer supplying 1–3 l per day will thus receive KSh 29.30–87.9 per day and KSh 879–2637 per month. There are no enrolled persons in this income group, which might mean that an income from milk sales below KSh2637 is insufficient to enrol. Of the enrolled group, 71% indicated that they supplied 4–6 l per day (KSh 117.2–175.8 per day / KSh3516 to 5274 per month).

Only 34% (*n* =45) of the enrolled and 27% (*n* = 32) of the non-enrolled respondents indicated that they were able to supply milk 10–12 months a year to TDPL. This means that the majority of the respondents were not able to supply milk continuously to the plant in the 12 months prior to the date that they were interviewed. Reasons for this might be that during the dry season, the cows give less milk, and during the calf-bearing period, the cows dry up. Consequently, TCHP members face interruptions in paying the premium via their milk account. This is therefore taken as one of the factors influencing enrolment in the TCHP, because TDPL members may indeed foresee their own inability to supply milk consistently throughout the year. This is a common phenomenon in sub-Saharan Africa where a large proportion of the population is actively involved with rain-fed agriculture. However, through enrolment in a CHI, payment is disassociated from the use of health services, creating a financial buffer between service fees and seasonal fluctuations [29]. Nevertheless, the financial buffer of the average TDPL member seems to be too small to bridge the gap of these seasonal fluctuations.

The results of this study indicated that travel time to health facility and enrolment are not significant. The findings of another study indicated that greater distance from the health facility was positively associated with enrolment in the CHI [24]. A similar finding in the analysis of the reasons for CHI dropout was noted as the researchers found that a shorter distance to the contracted health facility increased the dropout rate [30]. The reasons for this were verified by a qualitative study which revealed that those living far from the facility felt that they faced higher non-medical costs when seeking care, due both to the cost of transport and to the necessity of seeking such care [24]. They therefore came to value CHI as a tool to relieve them from at least part of the financial burden they faced when becoming ill or injured [30]. Yet, in another study it was found that the long distance to the closest health facility clearly correlated with low enrolment [26]. In this study five respondents also remarked in the open-ended question that the health facilities were far. This CHI only provides transport during emergencies.

The factors associated with enrolment were identified by applying a logistic regression model. In this model the quantity of supplied milk, which is an indicator of SES, was included. Milk supply was grouped as those supplying more than six litres and those supplying at best six litres per day. Furthermore, the model included a visit by a TCHP sales executive; information provision; and an understanding of CHI.

The adjusted effect of the quantity of daily milk supply was strongly associated with enrolment. This was notable and it meant that the TCHP was able to reach those farmers with low SES. Although the CHI concept was originally intended to reach the poorest of the poor, the practice of most CHI schemes showed that poor people were less likely to enrol [31]. This meant that the TCHP seemed to reach specifically the poor and the vulnerable, taking into account their financial situation, gender, or other factors that can hinder participation in the CHI [32].

Information provision was significantly associated with enrolment in the TCHP (OR: 8.77; 95% CI: 2.25–34.16). A unit increase in the score of information provision caused almost nine times increase in the odds of enrolment. The precision of this estimate, however, may be low because of the small sample size.

The remarks made by the respondents support the need for more education on CHI and TCHP. Attention should also be given to the affordability of CHI as 29.6% (*n* = 40) remarked that it was expensive.

### Limitations

The study was limited to one geographical area, namely the area around the TDPL milk collection centre of Lemook. The farmers of the other three TDPL milk collection centres were thereby excluded. The researchers conducted convenience sampling by interviewing farmers for one day at the market and for two days in their homesteads. Convenience sampling is considered a weak approach to sampling because it provides little opportunity to control for biases [13]. This could influence the findings of this study.

The sample size of 135 respondents was adequate to represent the target population. Of the sample 14% (*n* = 17) was enrolled to TCHP, which was representative of the enrolled percentage of the target population. However, the small number of 17 enrolled respondents endangered the power of the study. Power is the capacity of a study to detect differences or relationships that actually exist in the population [13]. Nevertheless significant differences and relationships were detected by conducting factor analysis and logistic regression.

Most of the interviews were conducted in the Kiswahili language. Although the interviewers internalised the translated version of the interview guide, translation and interpretation of the items might have influenced the responses to the research questions.

Another possible limitation is the cross-sectional design. This study took place during the dry season, which could influence the results of the study.

## Conclusion

It seems as if the TCHP could benefit a lot from improved marketing strategies. Information on the TCHP management should be provided in several accessible ways and that sales executives provide the target group with correct and complete information during visits.

Financial restrictions also have a prohibiting effect on TCHP. To make TCHP financially viable, it is recommended that workable solutions for social inclusion of the poorest and those with low income should be identified and that cost-effective ways be created in which farmers who are not able to supply milk consistently might be able to bridge the gap of the “dry” months in milk production.

This paper discussed the demographics of the respondents, data presentation, analysis and interpretation with the use of descriptive and inferential statistics. Descriptive statistics were presented in the form of percentages, frequencies, tables and pie charts. Inferential statistics were used to identify relationships between and among variables. Exploratory factor analysis was used to examine interrelationships among reasonably large variables and to disentangle them to identify clusters of variables that were linked together. Variables were finally reduced to two factors, namely “information provision” and “understanding CHI”. Logistic regression was conducted to identify the factors associated with enrolment in the TCHP. Farmers who supplied more than six litres of milk per day were less likely to be enrolled when compared to those supplying less than six litres per day. A visit by a TCHP sales executive increased the chances of enrolment, and information provision was strongly associated with enrolment in the TCHP.

## Additional file

Additional file 1: Table S1. Monthly number of milk suppliers (September 2011-August 2012). (DOCX 14 kb)

## Abbreviations

AAR: African Air Rescue
CHI: Community health insurance
CI: Confidence interval
KMO: Keiser-Meyer-Olkin
KSh: Kenyan Shilling
NHI: National Health Insurance
OR: Odd’s ratio
PCA: Principal components analysis
SES: Socio-economic status
TCHP: Tanykina Community Healthcare Plan
TDPL: Tanykina Dairy Plant Ltd
THE: Total health expenditure
WHO: World Health Organization
