# Supplementary material for: A quantitative study on factors influencing enrolment of dairy farmers in a community health insurance scheme
Source: BMC Health Serv Res. 2016 Dec 9;16:686. doi: 10.1186/s12913-016-1925-1 (PMC5148826; doi:10.1186/s12913-016-1925-1)
Supplement: Additional file 1: Table S1. — Monthly number of milk suppliers (September 2011-August 2012). (DOCX 14 kb) [file 12913_2016_1925_MOESM1_ESM.docx]

**Additional file 1**

**Table S1 Monthly number of milk suppliers (September 2011-August 2012)**

| **MONTH** | **PAID SUPPLIERS** |
| --- | --- |
| September 2011 | 4166 |
| October | 4647 |
| November | 4724 |
| December | 4642 |
| January 2012 | 4406 |
| February | 3308 |
| March | 2474 |
| April | 1948 |
| May | 2161 |
| June | 2577 |
| July | 2902 |
| August | 2434 |
| **Monthly average** | **3366** |
